# IL-36γ is secreted through an unconventional pathway using the Gasdermin D and P2X7R membrane pores

**DOI:** 10.3389/fimmu.2022.979749

**Published:** 2022-08-18

**Authors:** Laura D. Manzanares-Meza, Claudia I. Gutiérrez-Román, Albertana Jiménez-Pineda, Felipe Castro-Martínez, Genaro Patiño-López, Eunice Rodríguez-Arellano, Ricardo Valle-Rios, Vianney F. Ortíz-Navarrete, Oscar Medina-Contreras

**Affiliations:** ^1^ Mexico Children’s Hospital, Endocrinology, Epidemiology & Nutrition Research Unit, Mexico City, Mexico; ^2^ Department of Molecular Biomedicine, Center for Research and Advanced Studies, National Polytechnic Institute (CINVESTAV), Mexico City, Mexico; ^3^ Hospital Infantil de Mexico, Unidad de Investigación en Inmunología y Proteómica, Mexico City, Mexico; ^4^ Genomic Medicine Laboratory, Hospital Regional Lic. Adolfo López Mateos, Mexico City, Mexico; ^5^ Unidad Universitaria de Investigación, División de Investigación, Facultad de Medicina, Universidad Nacional Autónoma de México, Mexico City, Mexico

**Keywords:** IL-36γ, inflammation, macrophages, cytokin receptors, secretion

## Abstract

Mucosal innate immunity functions as the first line of defense against invading pathogens. Members of the IL-1 family are key cytokines upregulated in the inflamed mucosa. Inflammatory cytokines are regulated by limiting their function and availability through their activation and secretion mechanisms. IL-1 cytokines secretion is affected by the lack of a signal peptide on their sequence, which prevents them from accessing the conventional protein secretion pathway; thus, they use unconventional protein secretion pathways. Here we show in mouse macrophages that LPS/ATP stimulation induces cytokine relocalization to the plasma membrane, and conventional secretion blockade using monensin or Brefeldin A triggers no IL-36γ accumulation within the cell. *In silico* modeling indicates IL-36γ can pass through both the P2X7R and Gasdermin D pores, and both IL-36γ, P2X7R and Gasdermin D mRNA are upregulated in inflammation; further, experimental blockade of these receptors’ limits IL-36γ release. Our results demonstrate that IL-36γ is secreted mainly by an unconventional pathway through membrane pores formed by P2X7R and Gasdermin D.

## Introduction

Mucosal innate immunity functions as the first line of defense against invading pathogens. A key cellular component of these innate immune responses are macrophages (1). During infection and the subsequent inflammation, macrophages recognize Pathogen Associated Molecular Patterns (PAPs) and initiate downstream signaling that promotes the expression and secretion of inflammatory cytokines (2). Several members of the IL-1 family (IL-1α, IL-1β, IL-18, IL-33) are key cytokines upregulated in the inflamed mucosa (3).

This superfamily of cytokines is formed by eleven members, classified into three subfamilies: IL-1 subfamily, IL-18 subfamily and, IL-36 subfamily (4). The IL-36 subfamily has been recently described and involved in the development of several inflammatory diseases such as inflammatory bowel disease, rheumatoid arthritis, or psoriasis (5, 6).

The IL-36 subfamily consists of four members, a receptor antagonist (IL-36Ra), and three agonists, IL-36α, IL-36β and, IL-36γ (5). These agonists signal through the same receptor (IL-36R) (7) in a MyD88-dependent manner and activate NFκB and MAPK pathways; thus, leading to leukocyte recruitment and amplification of inflammation (8).

Inflammatory cytokines are regulated through their activation and secretion mechanisms to limit their function and availability (9), and cytokine processing is the first regulatory checkpoint (10). IL-1 cytokine superfamily requires processing to achieve full bioactivity (11). IL-36 cytokines are processed by neutrophil proteases (elastase, proteinase-3, and cathepsin G) (12) and macrophage- and epithelial cell-derived cathepsin S (13).

The second checkpoint corresponds to its secretion, and this mechanism is a fundamental response to damage and infection (14). IL-1 cytokines secretion is affected by the lack of a signal peptide on their sequence (10), which prevents them from accessing the conventional protein secretion pathway (CPS). Thus, they use unconventional protein secretion pathways (UPS) that involve membrane pores, vesicles, transporters, among others (15). IL-1β is associated with two types of unconventional secretory pathways (16), vesicles and membrane pores (17). The receptor P2X7R is involved in IL-1β secretion under ATP exposure, a stimulus that opens a pore and promotes vesicle shedding (18). Similarly, the membrane pore formed by Gasdermin D (GSDMD) has been involved in IL-1β secretion under inflammatory conditions (19). However, it remains unknown if members of the IL-36 family follow the same secretion mechanisms.

The activation and secretion checkpoints are of paramount importance as they have a direct impact on cytokine functionality, which could help in the development of new therapeutic targets. Since IL-36 cytokines participate in the development of several inflammatory and autoimmune diseases, and little is known about their function and signaling mechanisms, exploring the mechanisms that regulate the secretion of the IL-36γ cytokine is fundamental to further understand IL-36 cytokines biology.

We studied IL-36γ secretion, and we found that LPS induced expression of IL-36γ cytokine in IC21 macrophages and perinuclear localization, and LPS/ATP induced its secretion. We explored non-conventional secretion pathways and observed P2X7R or GSDMD pores blockade limits the cytokine release. Collectively, our results show that IL-36γ cytokine follows a non-conventional secretion pathway using P2X7R and GSDMD pores.

## Materials and methods

### Cell lines

Murine macrophages cell line IC21 (TIB-186) was cultured according to the manufacturer’s instructions (ATCC, VA).

### Cytokine induction

1x10^6^ IC21 cells/well were seeded in a 48-well plate (Corning, MA) and grown in RPMI-1640 (Corning, MA) medium supplemented with 10% fetal bovine serum (PAN Biotech, Germany), antibiotic/antimycotic (Corning, MA), and L-glutamine (Corning, MA). 24h after cell seeding the cells were stimulated for 18h with 1µg/ml LPS (Sigma-Aldrich, MO), and protein lysates were purified.

### Cytokine secretion

LPS-stimulated IC21 cells were co-stimulated for 1h with 3mM ATP (*In vivo*Gen, CA), in the presence or absence of 2 µM monensin (Biolegend, CA), 5 µg/ml brefeldin A (Biolegend, CA), 200nM A438079 hydrochloride (TOCRIS, UK) or 20µM Necrosulfonamide (NSA; TOCRIS, UK) (specific inhibitors specific for P2X7R and Gasdermin D respectively). Supernatants and protein lysates were evaluated after co-stimulation.

### Protein precipitation

The proteins present in the supernatant were precipitated with a 1:4 water-acetone mix at -20°C for 24h. The solution was centrifuged at 4700 g (Thermo-Fisher, MA) for 30min, and the pellet was washed twice with water-acetone and resuspended in RIPA (Sigma-Aldrich, MO).

### Western blot

40µg total protein were probed by western blot using primary Ab anti-IL-36γ TA505994; Origene, MD; 1:4,000), anti-GAPDH (GTX100118; GeneTex, CA; 1:1,000), and secondary anti-mouse-HRP (7076P2; Cell Signaling, MA; 1:5,000).

### Confocal microscopy

3.5x10^5^ IC21 cells were seeded in 25mm sterile coverslips (Corning, MA) in a six-well plate (Corning, MA) and were LPS-induced and ATP-stimulated as before. After stimulation, the cells were fixed with 4% paraformaldehyde, permeabilized with 0.2% Triton, blocked with 0.2% BSA in PBS, and stained with the rabbit primary antibody α-IL-36γ (TA326667; Origene, MD; 1:1000), followed by staining with the secondary Alexa Fluor 594 α-rabbit IgG antibody (8889S; Cell Signaling, MA; 1:2000) and DAPI (Sigma-Aldrich, MO). Images were acquired using a white light laser Leica TCS SP8 X confocal microscope at 60X (Leica, Wetzlar, Germany).

### *In silico* modeling

The amino acid sequence of mouse IL-36γ, P2X7R, and GSDMD were retrieved from UniProtKB (Q8R460, Q9D8T2, and Q9Z1M0, respectively). Models were built in the SWISS-MODEL server (20) using the crystals for human IL-36γ, rat P2X7R, and mouse GSDMD as templates, and the models with a Qmean score closest to 0 were accepted. The program MOE was used to refine the models using energy minimization and removal of water molecules, and the server SymmDock (21) was used to build the 16-subunit oligomer GSDMD pore.

### Statistical analysis

GraphPad Prism v9.3 software was used for data analysis. Statistical significance was determined by Student t-test between two groups. Mean ± SD of the data is presented. p<0.05 was statistically significant.

## Results

### LPS/ATP induces IL-36γ expression and secretion in IC21 macrophages

**Figure 1:**
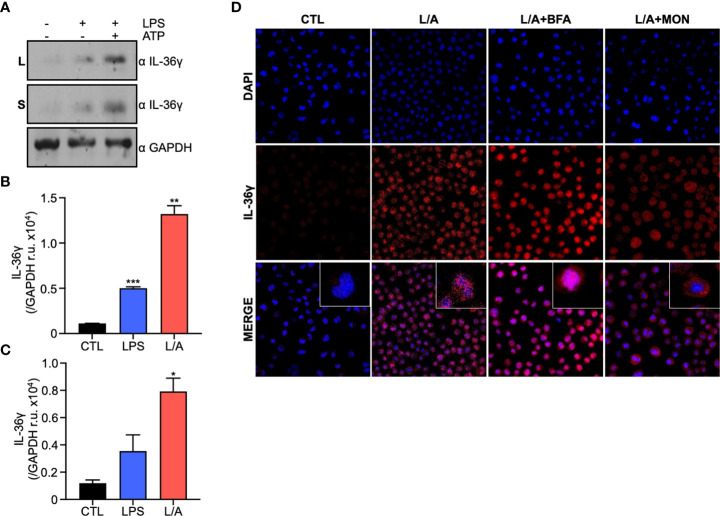
IL-36γ is secreted in response to ATP in a non-conventional pathway. **(A)** IL-36γ protein expression in lysates (L) and supernatants (S) of LPS or LPS/ATP activated macrophages. Densitometry of IL-36γ intensity in lysates **(B)** and supernatants **(C)** of cell stimulated with LPS or LPS/ATP (L/A). **(D)** IF of IC21 macrophages stimulated with LPS/ATP (L/A) and treated with monensin (Mon) or brefeldin A (BfA). *p<0.05, **p<0.01, ***p<0.001.

We first explored whether IL-36γ expression and secretion is altered by ATP, as other IL-1 superfamily cytokines (18). We stimulated IC21 murine macrophages and evaluated IL-36γ expression at protein (**Figures 1A, B** **)** and mRNA levels (data not shown). Stimulation with LPS increased IL-36γ expression, similar to previous reports in response to bacterial components (22). This correlates with the expression other members of the IL-36 subfamily under similar inflammatory conditions (23). We further observed that LPS/ATP stimulation induces the liberation of IL-36γ to the extracellular milieu (**Figures 1A, C** **)**. Taken together, these results suggests that bacterial components, like LPS and ATP, not only induce IL-36γ expression but also trigger its secretion.

### IL-36γ does not follow the conventional secretory pathway

Cytokine secretion to the extracellular milieu is of paramount importance during inflammation to coordinate defense mechanisms in the host. Eucaryotic cells use the conventional and unconventional protein secretion pathways. The conventional secretory pathway involves the translocation of proteins into the endoplasmic reticulum (ER), transport to the Golgi apparatus and finally to the plasma membrane for their secretion. This pathway depends on a signal peptide at the N-terminal portion of the protein to follow this route (24). As it has been hypothesized that IL-36γ lacks a signal peptide (11), we used the server signal IP 5.0 to predict the presence of a signal peptide *in silico* and confirmed there is no signal peptide predicted on IL-36γ amino acid sequence (data not shown). We then explored if IL-36γ follows the conventional secretory pathway using monensin (MN) and brefeldin A (BfA) to block secretion. MN is a Na^+^ ionophore that affects regions of the Golgi apparatus that are linked to the final stages of secretory vesicle maturation. BfA is a macrocyclic lactone produced by fungi that inhibits protein secretion from the ER to the Golgi apparatus (25). We observed that when MN or BfA are added to the cell culture, stimulation with LPS/ATP triggers no IL-36γ accumulation within the cell (**Figure 1D**), demonstrating that IL-36γ does not follow the conventional secretory pathway for its secretion.

### IL-36γ can pass through P2X7R and GSDMD *in silico*

**Figure 2:**
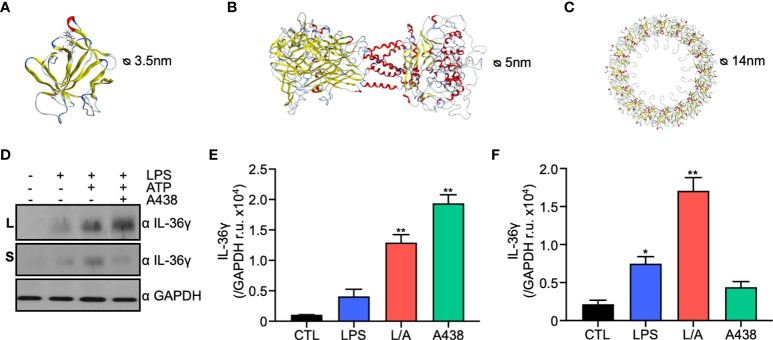
IL-36γ is secreted through the membrane pores P2X7R and GSDMD of the unconventional secretory pathway type I. **(A)** *In silico* model of mouse IL-36γ generated by SWISS-MODEL. **(B)** *In silico* model of P2X7R generated by SWISS-MODEL. **(C)** *In silico* model of GSDMD pore generated by SymmDock sever. **(D)** Western blot of lysates and supernatants under control, LPS, LPS/ATP (L/A), LPS/ATP+A438 conditions. Densitometry of IL-36γ in lysates **(E)** and supernatants **(F)**. *p<0.05, **p<0.01.

Lack of a signal peptide on IL-36γ sequence suggests it uses the unconventional secretory pathway. Of the four unconventional secretion pathways, membrane pores are the preferred mechanism for proteins that lack a signal peptide (15). In macrophages P2X7R (26) and GSDMD (19) form pores during inflammation or hyperactivation in an ATP-dependent manner. Both pores have been previously involved in IL-1β secretion (18, 19). Thus, we evaluated if IL-36γ, like IL-1β, uses P2X7R and/or GSDMD to exit the cell. We performed an *in silico* modeling of full-length and processed mouse IL-36γ, P2X7R, and GSDMD through three different servers, Swiss-Model, IntFold, and Robetta. The quality of the models was assessed through the Qmean score, Ramachandran score, and Molprobity score. The models built through the Swiss-Model server had the best scores and were used for further analysis. Full length (FL) and serin 42 processed (S42) IL-36γ were modeled based on the human IL-36γ crystal. The validation of the model gave a Qmean score of -0.53, Ramachandran score of 96.64% and Mol probity score of 1.29, indicating a good quality model. Processed IL-36γ model had the same scores as the FL variant, which suggests that the size reduction does not generate significant structural changes in the *in silico* model. Then, we analyzed the size and net charge, and we observed that both proteins have a positive net charge and an approximate diameter of 3.7nm (FL) and 3.5nm (S42) (**Figure 2A**). Next, we built the mouse homotrimer P2X7R in an open state, with bound ATP, based on the rat crystal due to the 85% identity. Validation of the model gave a Qmean score of -2.70, Ramachandran score of 94.79 and Molprobity score of 1.26. The pore diameter of P2X7R in an open state is 5nm in the *in silico* model, and has a negative net charge, particularly in the transmembrane and extracellular portions of the receptor (**Figure 2B**). Next, we built the NT-GSDMD model since it is the fraction that oligomerizes and forms the membrane pore. The model was generated based on the mouse crystal of full GSDMD due to the 97.97% identity. We obtained a medium-low quality model with a Q-mean score of-4.73, Ramachandran score of 86.67%, and Molprobity of 2.86. This low quality could be due to the low resolution of the crystal in the NT portion of the protein. We built the 16nm oligomer through the server SymmDock, which generates a 14nm diameter pore (**Figure 2C**). According to the data obtained by our *in silico* models, 3.7 and 3.5nm positively charged IL-36γ can theoretically pass through both the 5nm P2X7R pore and the 14nm GSDMD pore.

Consistent with these results, we observed P2x7r and Gsdmd are upregulated in a microarray from pro-inflammatory intestinal mouse macrophages (**Figure S1A**) as well as LPS-activated IC21 macrophages (**Figure S1B**), suggesting an increased bioavailability in conditions where IL-36 γ is expressed.

### IL-36γ is secreted through membrane pores of the unconventional protein secretion pathway under inflammatory conditions

To confirm the *in silico* data, we evaluated IL-36γ secretion through P2X7R and GSDMD experimentally. First, we used the P2X7R specific inhibitor A438 that binds to the inner face of the receptor blocking its opening, even in the presence of ATP. The presence of IL-36γ was evaluated by western blot in lysates and supernatants of IC21 macrophages under LPS or LPS/ATP and A438. We observed that in the presence of a P2X7R specific inhibitor, IL-36γ is accumulated within the cell, as observed in the lysates (**Figures 2D, E**). In contrast, when we evaluated the supernatants, we observed a clear reduction in secreted IL-36γ of approximately 75% (**Figures 2D, F**), suggesting that P2X7R participates in IL-36γ secretion. Then, we evaluated IL-36γ secretion by GSDMD pore. We used the specific inhibitor Necrosulfonamide (NSA), which binds to the NT domain of processed GSDMD and prevents its oligomerization and pore formation in the plasma membrane. Similar to P2X7R, we observed that in the presence of NSA, IL-36γ is accumulated within the cell (**Figure S1C**), suggesting that GSDMD also participates in IL-36γ secretion. Our data demonstrate that P2X7R and GSDMD have a role in IL-36γ secretion.

### IL-36γ localizes to the perinucleus of activated IC21 macrophages

**Figure 3:**
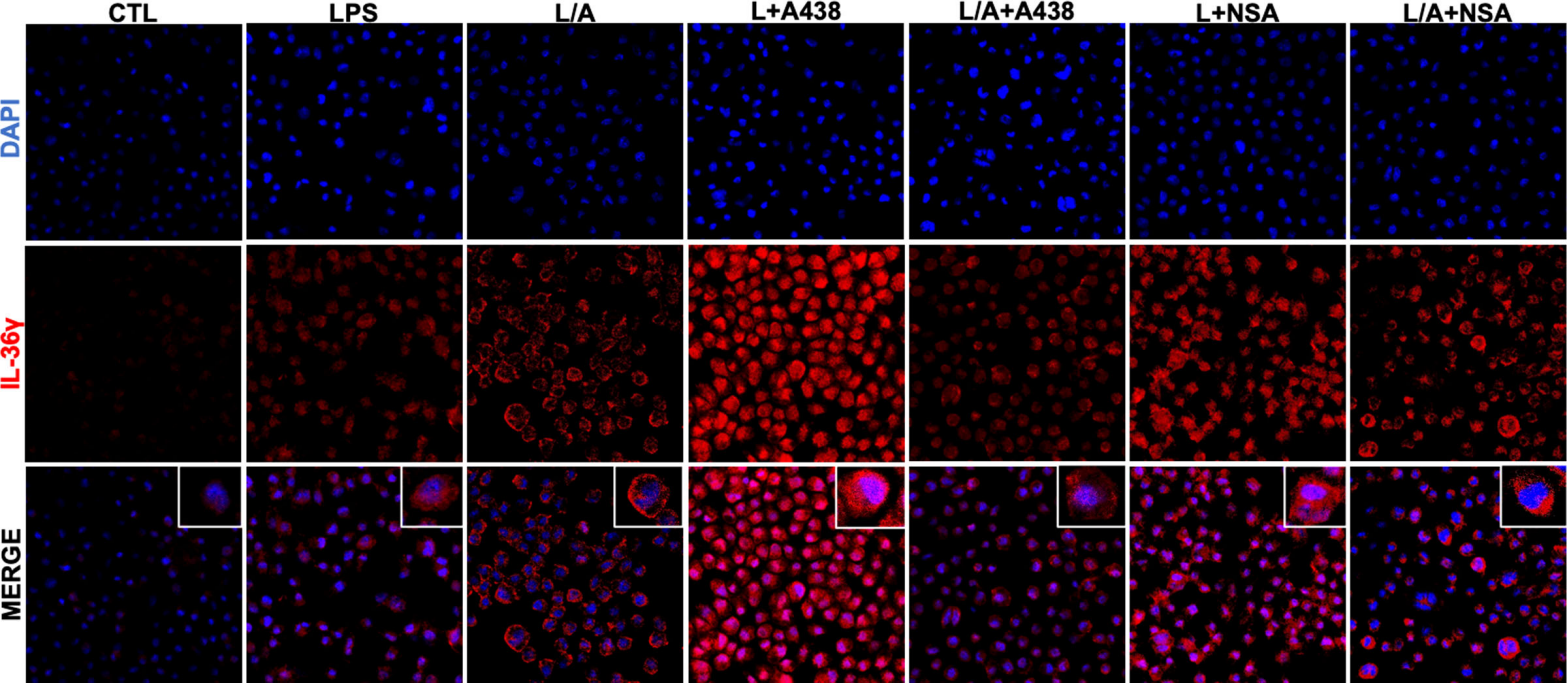
IL-36γ is localized in the cytoplasm of IC21 macrophages. IL-36γ localization in IC21 macrophages in control, LPS, LPS/ATP (L/A), LPS/ATP+A438, and LPS/ATP+NSA conditions. nuclei staining (DAPI), IL-36γ (Alexa Fluor594).

Finally, we evaluated IL-36γ localization within the cell in CTL, LPS, LPS/ATP, and LPS/ATP+A438/NSA conditions (**Figure 3**). We observed that IL-36γ localizes in the cytoplasm of IC21 macrophages, particularly in a perinuclear fashion, in CTL and LPS conditions. In the presence of LPS/ATP we observed that IL-36γ is accumulated in a scatter pattern in the cytoplasm and close to the plasma membrane, which falls in line with the induction of its secretion by the same stimulus, showing that LPS/ATP-induced secretion mobilizes IL-36γ towards the plasma membrane. In contrast, when we added the specific inhibitors A438 or NSA, IL-36γ changes its location and goes back to the perinuclear region as observed in CTL and LPS conditions. In contrast, when we added the specific inhibitor NSA, IL-36γ localizes close to the plasma membrane. Thus, our results show that the inhibition of IL-36γ secretion through P2X7R prevents the cytokine relocalization within the cell.

Together, our results demonstrate that IL-36γ is secreted mainly by an unconventional pathway through membrane pores formed by P2X7R and GSDMD.

## Discussion

In recent years there has been a growing interest in IL-36γ function and its involvement in the development of inflammatory diseases. However, a few mechanisms in this cytokine biology remain unknown. IL-36γ lack of a signal peptide (8, 22) suggests it could have access to unconventional secretory pathways (15, 27). Our data shows that the addition of LPS/ATP induces IL-36γ secretion. Our data agree with previous reports that have established that secretion of some members of the superfamily of IL-1, such as IL-1β, IL-18, and IL-36α, need two signals. LPS works as the first signal as it induces the expression of the cytokines; meanwhile, ATP works as the second signal triggering the secretion of the cytokines (23). It is interesting that such stimulation also activates P2X7R, an ATP receptor, as well as the inflammasome, which in turn activates GSDMD. GSDMD, once it is cleaved by caspase 1, oligomerizes and forms a pore on the plasma membrane (26, 28). Both P2X7R and GSDMD are members of the unconventional secretory pathway type I (15). Thus, we decided to evaluate if IL-36γ, like IL-1β and IL-18 (17, 29), is also secreted through these membrane pores. First, we evaluated *in silico* the IL-36γ secretion capabilities. According to our generated 3D models, the 3.7nm and 3.5nm size (full length and processed respectively), as well as the net positive charge of IL-36γ, allow its passage through the 5nm P2X7R and the 14nm GSDMD pores. Experimentally, we observed a significant intracellular accumulation and an extracellular reduction in IL-36γ levels when both P2X7R and GSDMD pores are blocked, confirming IL-36γ uses an unconventional secretion pathway. Our data suggest that secretion through membrane pores of the unconventional secretory pathway type I could be a common secretion mechanism for cytokines of the IL-1 superfamily. Consistent with our results, it has been observed that IL-36α is also secreted through P2X7R (23).

IL-36γ localizes preferentially to the cytoplasm of IC21 macrophages, particularly in a perinuclear region under homeostatic conditions. After LPS stimulation it remains localized to the perinuclear region, but LPS/ATP scattered IL-36γ in the cytoplasm closer to the plasma membrane, which agrees with its secretion in response to this stimulus. Other members of the superfamily like IL-1α, IL-1β, and IL-18 have polybasic regions in their sequence, which allows them to adhere to the plasma membrane in preparation for secretion (16); we found through *in silico* prediction that IL-36γ contains a small polybasic region that could allow its association to the plasma membrane through electrostatic interactions. Also, we observed that under the P27XR specific inhibition, IL-36γ appears to go back to the perinuclear area. In contrast, under GSDMD specific inhibitor IL-36γ remains close to the membrane. Suggesting that ATP is the stimuli that affects IL-36γ localization since we only observed this phenomenon when P2X7R is blocked. Previous studies have shown that IL-33, member of IL-1 superfamily, localizes to the nucleus under homeostatic conditions and it relocalizes to the cytoplasm in response to stress. This relocalization depends on the microtubule network and ATP (30). It has been shown that ATP participates in microtubule assembly (31) and it triggers vesicle movement towards the plus end for their secretion (32). Thus, IL-36γ localization dependance on ATP could be explained if IL-36γ travels along microtubules to be secreted. It’s worth exploring if IL-36γ is intracellularly localized in vesicles that could potentially travel along microtubules towards the plasma membrane for its exocytosis.

Here we have demonstrated that IL-36γ is secreted through an unconventional secretory pathway type I, specifically through the P2X7R and GSDMD membrane pores in a mechanism dependent on LPS/ATP stimulation. As P2X7R/GSDMD have an important role in the secretion of IL-36γ; thus, we believe that blocking these pores can be a novel therapeutic approach to limit IL-36 cytokines bioavailability in several autoimmune diseases, cancer, obesity, and chronic inflammatory pain, among others, and ameliorate symptoms, as has been proposed elsewhere (33, 34).

## Data availability statement

Publicly available datasets were analyzed in this study. This data can be found here: http://www.ncbi.nlm.nih.gov/geo/query/acc.cgi?token=olgvmwkanfgzlcd&acc=GSE68269.

## Author contributions

OM-C and LM-M conceived and designed the analysis. LM-M, CG-R, AJP and FC-M collected the data. GP-L, ER-A, RV-R and VO-N contributed data or analysis tools. LM-M and OM-C performed the analysis. OM-C and LM-M wrote the paper. All authors contributed to the article and approved the submitted version.

## Funding

This work was supported by CONACYT grant CB2016-01/280815 to O. Medina-Contreras. L Manzanares-Meza received the CONACYT scholarship 589088.

## Acknowledgments

We thank D. Castro-Eguiluz for her scientific editing and writing support.

## Conflict of interest

The authors declare that the research was conducted in the absence of any commercial or financial relationships that could be construed as a potential conflict of interest.

## Publisher’s note

All claims expressed in this article are solely those of the authors and do not necessarily represent those of their affiliated organizations, or those of the publisher, the editors and the reviewers. Any product that may be evaluated in this article, or claim that may be made by its manufacturer, is not guaranteed or endorsed by the publisher.

## Supplementary material

Supplementary Figure 1
**(A)** Microarray analysis from DSS-treated mouse colonic macrophages. **(B)** qPCR expression for P2x7r and Gsdmd in IC21 macrophages stimulated with LPS or LPS/ATP. **(C)** Densitometry of IL-36γ in macrophages under CTL, LPS/ATP or LPS/ATP+NSA stimulation.
