# Supplementary figures and images for: IL-36γ is secreted through an unconventional pathway using the Gasdermin D and P2X7R membrane pores
Source: Front Immunol. 2022 Aug 18;13:979749. doi: 10.3389/fimmu.2022.979749 (PMC9434278; doi:10.3389/fimmu.2022.979749)

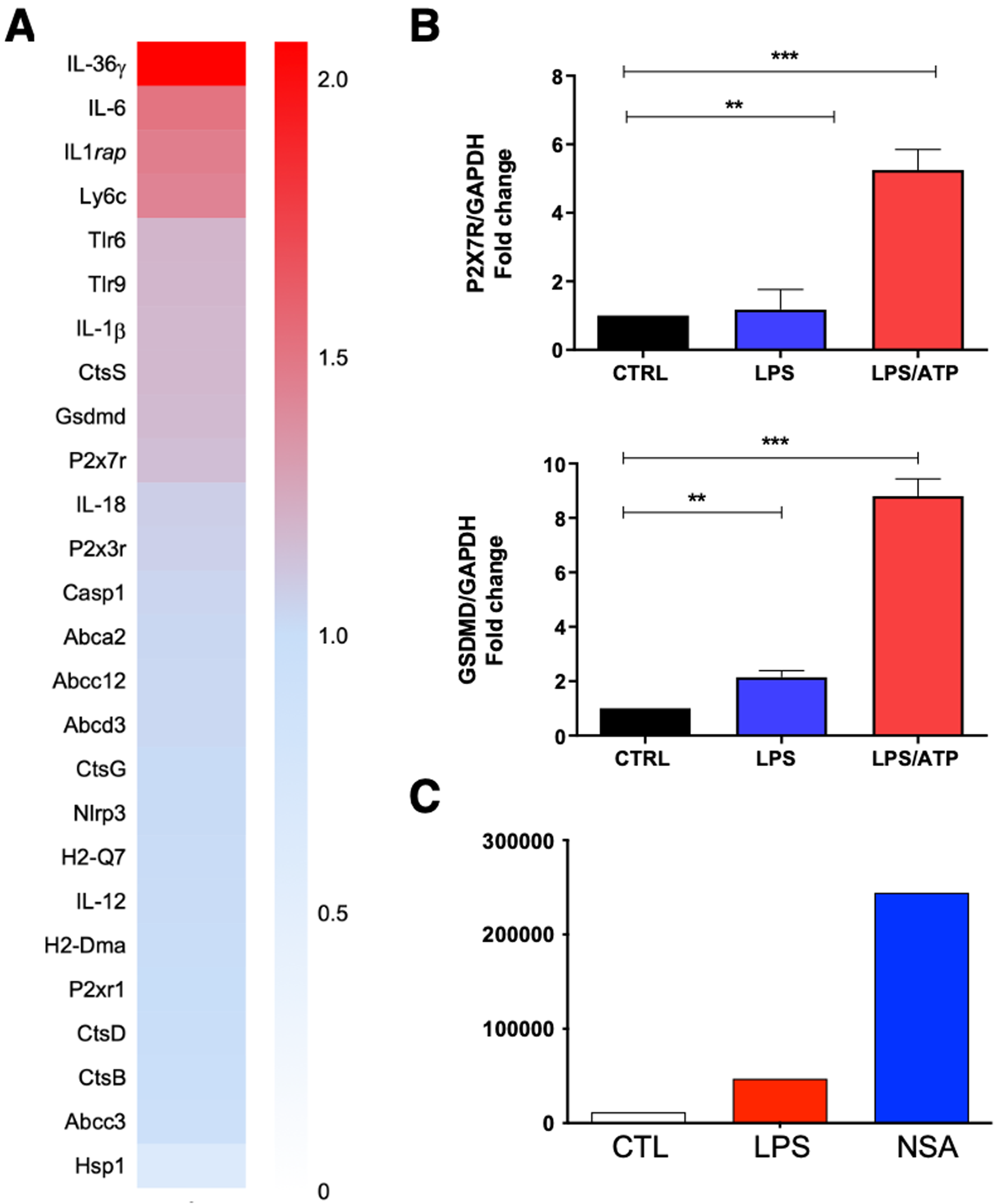

Supplement: Supplementary Figure 1 — (A) Microarray analysis from DSS-treated mouse colonic macrophages. (B) qPCR expression for P2x7r and Gsdmd in IC21 macrophages stimulated with LPS or LPS/ATP. (C) Densitometry of IL-36γ in macrophages under CTL, LPS/ATP or LPS/ATP+NSA stimulation. [file Image_1.tif]
